# Shared *Escovopsis* parasites between leaf-cutting and non-leaf-cutting ants in the higher attine fungus-growing ant symbiosis

**DOI:** 10.1098/rsos.150257

**Published:** 2015-09-30

**Authors:** Lucas A. Meirelles, Scott E. Solomon, Mauricio Bacci, April M. Wright, Ulrich G. Mueller, Andre Rodrigues

**Affiliations:** 1 Department of Biochemistry and Microbiology, UNESP—São Paulo State University, Rio Claro, São Paulo, Brazil; 2 Center for the Study of Social Insects, UNESP—São Paulo State University, Rio Claro, São Paulo, Brazil; 3 Department of Biosciences, Rice University, Houston, TX, USA; 4 Department of Integrative Biology, University of Texas at Austin, Austin, TX, USA

**Keywords:** ancestral state reconstruction, attine ants, host–parasite interactions, phylogeny

## Abstract

Fungus-gardening (attine) ants grow fungus for food in protected gardens, which contain beneficial, auxiliary microbes, but also microbes harmful to gardens. Among these potentially pathogenic microorganisms, the most consistently isolated are fungi in the genus *Escovopsis*, which are thought to co-evolve with ants and their cultivar in a tripartite model. To test clade-to-clade correspondence between *Escovopsis* and ants in the higher attine symbiosis (including leaf-cutting and non-leaf-cutting ants), we amassed a geographically comprehensive collection of *Escovopsis* from Mexico to southern Brazil, and reconstructed the corresponding *Escovopsis* phylogeny. Contrary to previous analyses reporting phylogenetic divergence between *Escovopsis* from leafcutters and *Trachymyrmex* ants (non-leafcutter), we found no evidence for such specialization; rather, gardens from leafcutters and non-leafcutters genera can sometimes be infected by closely related strains of *Escovopsis*, suggesting switches at higher phylogenetic levels than previously reported within the higher attine symbiosis. Analyses identified rare *Escovopsis* strains that might represent biogeographically restricted endemic species. Phylogenetic patterns correspond to morphological variation of vesicle type (hyphal structures supporting spore-bearing cells), separating *Escovopsis* with phylogenetically derived cylindrical vesicles from ancestral *Escovopsis* with globose vesicles. The new phylogenetic insights provide an improved basis for future taxonomic and ecological studies of *Escovopsis*.

## 1. Introduction

Symbiotic relationships are a significant source for evolutionary innovation [1–3], generating diversity in mutualistic and parasitic interactions, particularly among microbe–insect associations. During the last decades, understanding of microbe–insect interactions has significantly improved, revealing more complex interactions among an increasingly diverse set of participants than was previously appreciated. This is especially true for attine ants and their microbial symbionts, which have become among the best-studied examples for parasitic and mutualistic insect–microbe coevolution [4,5].

All known attine ants (more than 250 described species within 16 genera) cultivate basidiomycetous fungi as the primary source of food [6,7]. Since the origin of the symbiosis approximately 67 million years ago, five distinct types of fungiculture evolved [8–10]. We focus here on the phylogenetically derived higher attine fungiculture involving four ant genera: *Trachymyrmex*, *Sericomyrmex*, *Acromyrmex* and *Atta* (the last two genera comprise the leaf-cutting ants, the behaviourally most complex group within the entire tribe [9,10]). Higher attine ants cultivate phylogenetically derived fungi that exhibit hyphal swellings, called gongylidia, which are specialized structures used to feed the queen and the brood [11–13].

The gardens grown by attine ants are susceptible to invasion by fungi; some of these are only known from associations with attine ant nests and are considered specialized parasites [14] grouped within the genus *Escovopsis* [14–21]. Recently, a new closely related genus, *Escovopsioides*, was described, but the parasitic nature of this fungus is unknown [20].

Previous research argued that *Escovopsis* coevolved with attine ants and their cultivars [17], resulting in a clade-to-clade correspondence where different clades of the *Escovopsis* parasite would be specifically associated with distinct clades of ants and their corresponding fungi. For example, a monophyletic clade of three *Escovopsis* strains was found infecting *Trachymyrmex* gardens from Panama, Ecuador and Trinidad, suggesting that a specific clade of *Escovopsis* infects the *Trachymyrmex* symbiosis [17]. Based on the three strains examined (each from a single nest from each of these locations), the *Trachymyrmex*-associated *Escovopsis* was thought to be distinct from *Escovopsis* infecting leaf-cutting ants (*Acromyrmex* and *Atta*). However, to our knowledge to date no study has extensively explored the geographical diversity of *Escovopsis* associated with *Trachymyrmex*, especially in South America, the centre of diversity for all genera of higher attines. Studying *Escovopsis* strains from leaf-cutting ants, Taerum *et al*. [22,23] identified three main, monophyletic groups among leafcutter-associated *Escovopsis*, all of which were found in South and Central America. However, Taerum *et al*. [22,23] studied mostly *Escovopsis* collected in Panama (some samples from Argentina, Ecuador and one sample from Guiana and Mexico), but few representatives from across South America. Augustin *et al*. [20] studied *Escovopsis* strains associated with *Acromyrmex* species within a small geographical area in Brazil (Viçosa, Minas Gerais state) and showed that, even within a restricted area, three new species of *Escovopsis* were found infecting gardens of *Acromyrmex* ants. This previously unknown diversity uncovered in the small sample of Augustin *et al*. [20] suggests that South America might hold a significant unexplored diversity of *Escovopsis*.

Using an expanded collection of *Escovopsis* spanning almost the entire range of higher attine ants, including Brazil (*n*=55 strains), Argentina (*n*=1), Panama (*n*=13), Lesser Antilles (*n*=2), Trinidad and Tobago (*n*=1) and Mexico (*n*=3), we test here whether *Escovopsis* isolated from leaf-cutting colonies group separately from those isolated from *Trachymyrmex* colonies. Analysing *Escovopsis* strains isolated from 70 higher attine colonies (26 *Atta* colonies, 25 *Acromyrmex*, 18 *Trachymyrmex*, one *Sericomyrmex*), from at least 20 ant species in 31 locations, we show that leafcutter ants and other higher attine ants can share similar *Escovopsis* parasites, indicating no strict ant-parasite co-cladogenesis within this group of ants. We also identify several new *Escovopsis* strains forming previously unknown clades, which may correspond to new species. Correlations between morphological traits and phylogenetic patterns reveal a need for further taxonomic and ecological studies on these newly identified fungal pathogens.

## 2. Material and methods

### 2.1 Collection, isolation and morphological aspects

From 2001 to 2013, we sampled *Escovopsis* from Brazil (22 sites), Panama (four sites), the Caribbean island of Guadeloupe (one site), Argentina (one site) and Mexico (one site). To these collections, we added DNA sequence data from published studies of *Escovopsis* collected in Trinidad and Tobago by Seifert *et al*. [16] and in Viçosa, Brazil by Augustin *et al*. [20]. The combined dataset contains samples from 31 collection sites spanning the known geographical range of *Escovopsis* (see the electronic supplementary material, table S1, for complete information).

To isolate *Escovopsis*, pieces of attine fungus garden (one to five pieces with 3–5 cm^3^ each, depending on the size of the chamber) along with some worker ants were collected *in situ* from live ant colonies using a sterile spatula. Fungus gardens were maintained in ultraviolet-sterilized plastic containers until they reached the laboratory. Small fragments of the fungus garden (0.5–1 mm in diameter) were plated on potato dextrose agar (PDA) supplemented with chloramphenicol (150 mg l^−1^, Sigma) and incubated at 25^°^C in the dark for the following 14 days. Plates were checked twice daily for fungal growth and were subcultured on new PDA plates when *Escovopsis* growth became visible. To confirm the purity of *Escovopsis* cultures, monosporic cultures were obtained for each strain after isolation. Some strains were isolated from middens, but the isolation process was the same as described above (see the electronic supplementary material, table S1). We obtained 61 new *Escovopsis* strains from our survey and also analysed nine *Escovopsis* strains isolated by our research group and superficially characterized in previous studies [24,25]. Moreover, we included morphological data derived from five strains for brown-spored *Escovopsis* species previously identified [16,20]. Therefore, our morphological analysis comprised 75 *Escovopsis* strains associated with higher attine ants: 55 from Brazil, 13 from Panama, three from Mexico, two from the Caribbean island of Guadeloupe, one from Argentina and one from Trinidad and Tobago. *Escovopsis* strains RS019, RS020, RS030, RS053, RS055, RS061 and RS076 were superficially analysed by Rodrigues *et al*. [24], while strains NL007 and SES005 were used in *in vitro* bioassays by Meirelles *et al.* [25].

In addition to the 75 strains, we also analysed the morphology of five brown-spored *Escovopsis* strains isolated from *Apterostigma* by Gerardo *et al*. [19]; these strains were included mainly for comparison with our strains associated with higher attines. *Escovopsis* sampled from Brazil are stored in 10% glycerol at −80^°^C at UNESP—Microbial Resource Center in Rio Claro, SP, Brazil, while *Escovopsis* obtained from other countries are stored under the same conditions in the Section of Integrative Biology—University of Texas at Austin, Austin, TX, USA.

We performed morphological examinations on all new strains. For this purpose, the strains were grown in PDA at 25^°^C in the dark and, after 7 days of growth, microscopic slides were prepared to analyse the vesicle shape (globose or cylindrical).

### 2.2 DNA extraction, PCR and sequencing

Using a modified CTAB method [18,26], we extracted genomic DNA directly from colony mycelia of all strains after 7 days of growth in PDA. Previous studies used elongation factor 1-alpha (*tef1*) to reconstruct *Escovopsis* phylogenies [17–19,22,23]. Augustin *et al*. [20] showed the potential of using the internal transcriber spacer (ITS) as a phylogenetic marker for this fungus; this region is now considered the ‘barcoding region’ for fungi [27]. To increase our phylogenetic signal, we used both *tef1* and ITS for phylogenetic analyses.

For *tef1* amplification, we used primers derived from Taerum *et al*. [22]. The forward primer was the same EF6–20F (5^′^-AAGAACATGATCACTGGTACCT-3^′^); however, we modified one base on the reverse primer (‘R’ was replaced by ‘A’) and the final primer was EF6**A**-1000R (5^′^-CGCATGTC**A**CGGACGGC-3^′^). This change increased the melting temperature of the primer, minimized co-amplification and improved the sequencing results. DNA was diluted 1000–10 000 × (final concentration ≅0.5–5 ng μl^−1^) and the PCR conditions were: 96^°^C for 3 min, 35 cycles at 96^°^C for 30 s, 61^°^C for 45 s and a final extension step at 72^°^C for 1 min following Meirelles *et al*. [25].

ITS amplification was performed using primers ITS1 (5^′^-TCCGTAGGTGAACCTGCGG-3^′^) and ITS4 (5^′^-TCCTCCGCTTATTGATATGC-3^′^). DNA was diluted 100× (final concentration ≅ 50–100 ng μl^−1^) and the PCR conditions were: 96^°^C for 3 min, 35 cycles at 94^°^C for 1 min, 55^°^C for 1 min and a final extension step at 72^°^C for 2 min [28]. Owing to the high CG content in ITS for *Escovopsis* (more than 65% in some strains), we prepared reactions with 5% dimethyl sulfoxide to enhance primer annealing.

PCR products were cleaned up with Kit Wizard^®^ SV Gel and PCR Clean-up System (Promega) and the cycle sequencing reactions were prepared with 15–20 ng of template using BigDye^®^ Terminator v. 3.1 Cycle Sequencing Kit (Life Technologies) following the manufacturer’s instructions. Sequences were generated at UNESP using ABI3500 (Life Technologies) and in a DNA sequence facility at UT-Austin. Contigs were assembled using Bioedit v. 7.1.3 [29]. Sequences of all strains were deposited in GenBank under accessions KM817043–KM817173 (see the electronic supplementary material, table S1).

### 2.3 Phylogenetic analysis

We included in our analysis all the 61 new strains derived from this study as well as nine *Escovopsis* strains from Brazil used in previous studies [24,25], all associated with higher attine ants (see the electronic supplementary material, table S1). Moreover, sequences of five brown-spored *Escovopsis* strains associated with *Apterostigma* ants [19] were included along with sequences from described species for *Escovopsis* and *Escovopsioides* [16,20,21]. Finally, three other Hypocreaceae fungi used as an outgroup by Meirelles *et al*. [21] were maintained here. The final dataset comprised two files, one for *tef1* with 85 taxa and the other for ITS with 80 taxa (for the five *Escovopsis* strains derived from Gerardo *et al*. [19], only *tef1* but no ITS sequences were available). Alignments were obtained independently for each file using MAFFT v. 7 [30]. Nucleotide substitution models were selected under separated runs for each file using the Akaike and Bayesian information criteria (AIC) with a confidence interval of 95% in jModelTest 2 [31]. The phylogeny was reconstructed by two different methods: maximum likelihood (ML) using RAxML v. 8 [32] and Bayesian Inference (BI) using MrBayes v. 3.2.2 [33].

**Figure 1.**
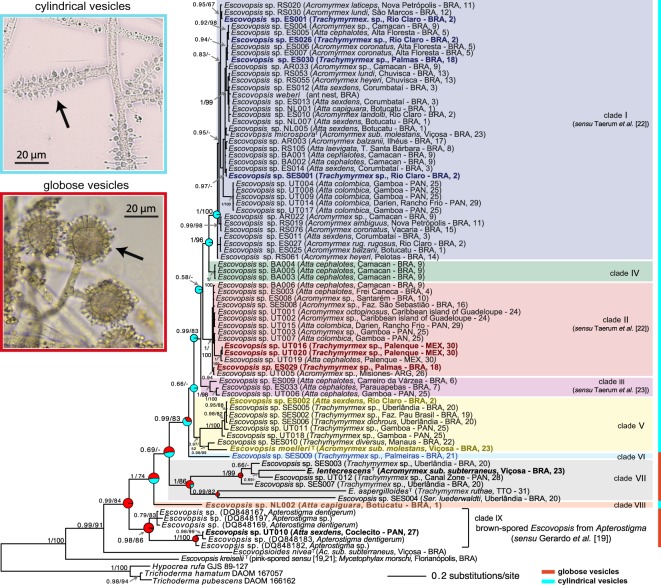
Phylogeny of *Escovopsis* associated with higher attine ants based on elongation factor 1-alpha (*tef1*) and internal transcribed spacer (ITS) markers analysed under Bayesian criteria. Pie-graphs at key nodes show probabilities of ancestral character-states for globose (red) and cylindrical (blue) vesicles. Coloured bars on the right side indicate the observed vesicle morphology, and black arrows point to the respective vesicle structure (400×). The main clades are highlighted in different colours and were consistently supported in both ML and Bayesian analyses. The phylogenetic tree includes also strains from brown-spored *Escovopsis* associated with *Apterostigma* ants [19], *Escovopsioides* *nivea* and *Escovopsis kreiselii* (the only species described for the pink-spored *Escovopsis*, a less derived group that infects lower attine ant gardens, [21]). Three additional species of Hypocreaceae fungi were used as an outgroup. *Escovopsis* strains in bold indicate exceptions of the co-cladogenesis model (see text). The ant species from which an *Escovopsis* strain was isolated, its corresponding collection sites and identification (ID) numbers are given in parentheses. GenBank accession number for *tef1* and ITS of each strain are available in the electronic supplementary material, table S1. Only posterior probabilities and bootstrap values greater than or equal to 0.5 or 50 are shown. The letter ‘T’ indicates ex-type strains (Ser., *Sericomyrmex*; *sub., subterraneus*; ARG, Argentina; BRA, Brazil; MEX, Mexico; PAN, Panama; TTO, Trinidad and Tobago).

We performed individual phylogenies for each marker, and the general phylogenetic topology was similar (data not shown) with respect to the well-supported clades and key features emphasized in the below discussion (e.g. despite the few topological differences within specific clades, both markers showed the same *Escovopsis* sharing between leaf-cutting and non-leaf-cutting ants). Our main analyses here therefore focus on the concatenated data (figure 1) combining information from both markers. For *Escovopsis* from higher attines, ITS is a phylogenetically informative marker, but *tef1* provides greater phylogenetic resolution.

The two different alignments were concatenated using Winclada v. 1.00.08 [34]. ML analysis was conducted in RAxML using GTR+I+G for each partition; 100 independent ML trees were reconstructed and the one with the best score was kept. To evaluate the tree reliability, 2000 bootstraps replicates were performed using the same model (bootstraps values converged at 1100 replicates [35]). For BI, GTR+I+G was also used as initial model for each partition. Two independent runs were performed; for both, three heated chains and one cold chain was used; each run consisted of Markov Chain Monte Carlo (MCMC) sampling for two million generations. Convergence occurred when the standard deviation of split frequencies fell below 0.01 and the first 25% of MCMC generations were discarded as ‘burn-in’. The final Bayesian tree was edited in FigTree v. 1.4.0 (http://tree.bio.ed.ac.uk/software/figtree/) and Adobe Illustrator CS6 (Adobe Systems) for final polishing.

### 2.4 Ancestral state reconstruction

One important microscopic morphological character for *Escovopsis* is the shape of their vesicles, which has been used in the taxonomic descriptions of new species [15,16,20]. *Escovopsis* vesicles are structures supporting spore-bearing cells (phialides) and two vesicle types can be distinguished, globose and cylindrical (figure 1). Because *Escovopsis* with globose vesicles grouped in less derived clades, whereas *Escovopsis* with cylindrical vesicles grouped in a derived clade, globose vesicles appeared to be the ancestral state. To test this hypothesis, we performed ancestral state reconstruction. Ancestral state inference using a phylogenetic tree assumes a single tree that is known without error. However, heuristic searches for phylogenetic tree estimation do not guarantee the discovery of the true tree topology and branch lengths. Therefore, we performed ancestral state reconstruction using the Ape package in R [36,37] over the ML topology estimated in RAxML, the Bayesian consensus topology estimated in MrBayes, a sample of trees from the Bayesian posterior sample and a sample from the RAxML bootstrap replicates to assess the robustness of our conclusions to slight variations in topology and/or branch lengths.

Ape incorporates multiple models that can be used for ancestral state reconstruction. We performed model fitting using likelihood ratio tests to determine whether a simple equal rates model could be rejected in favour of a more complex model, in which the rate of changing from globose to cylindrical vesicles differed from the rate of changing from cylindrical to globose vesicles. With an average *p*-value of 0.749, we could not reject the simpler model in favour of the more complex model for any tree in our sample; therefore, we performed the analysis under the equal rates model.

## 3. Results

*Escovopsis* strains derived from higher attine ants grouped within nine different clades, and four of them (I, II, V and VII) contain both *Escovopsis* from leaf-cutting ants and *Trachymyrmex* ants (figure 1). Representative strains from these four clades were isolated from multiple points in Brazil, Panama, Argentina, Mexico, Guadeloupe and Trinidad and Tobago (figure 1). Clades I and II consist mainly of *Escovopsis* isolated from leaf-cutting ants, while clades V and VII consist mostly of *Escovopsis* derived from *Trachymyrmex*, but we also found multiple exceptions to this clade-to-clade correspondence. Specifically, 12 of the 75 *Escovopsis* strains (16%) isolated from higher attine ants did not follow this pattern (figure 1). These results show that *Escovopsis* infecting leaf-cutting ants and those infecting other higher attine ants are not reciprocally monophyletic, suggesting that these insects can share infections caused by closely related strains of the parasite.

**Figure 2.**
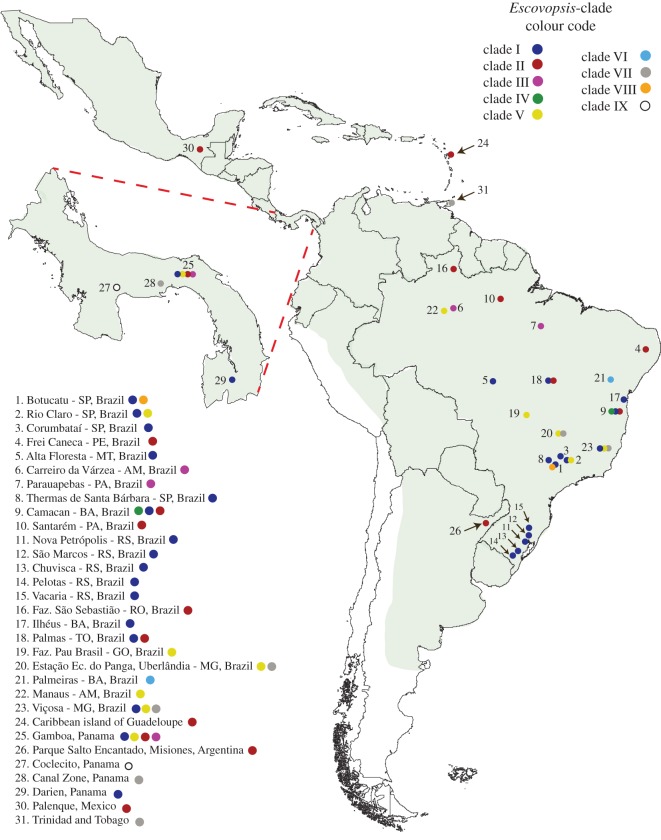
Geographical distribution of *Escovopsis* sampling sites. Coloured circles correspond to clade colours in figure 1. Location number corresponds to the listing in the electronic supplementary material, table S1. For sites from which different clades of *Escovopsis* were sampled, more than one coloured circle is shown, but this does not indicate that different *Escovopsis* strains derived from the same colony (see the electronic supplementary material, table S1 for nests IDs). The green shading indicates the approximate distribution of higher attine ants [6,38]. Cartographic data (original map) were obtained using a free package for the software DIVA-GIS [39].

This is, to our knowledge, the first study to report the occurrence of clades IV, V, VI and VIII. These results indicate that the phylogenetic diversity of *Escovopsis* is much greater than what was known. This is probably owing to the inclusion of samples from a geographical range unexplored before, especially from Brazil. Our results suggest that clades IV, VI and VIII may consist of rare *Escovopsis* types, as strains from these clades were each found in only one locality (figure 2). Clade IV consists of three phylogenetic identical strains isolated from two different *Atta cephalotes* colonies in Camacan, Bahia—BRA (site no. 9); despite possible abundance at this site, representatives of this clade have not been isolated at any other collection site (figures 1 and 2) suggesting the possibility of an endemic *Escovopsis* strain from this region.

As demonstrated by Taerum *et al*. [22], there was no correlation between leaf-cutting ant genera and particular *Escovopsis* strains. *Atta cephalotes* populations from Camacan were infected by *Escovopsis* from clades I, II and IV. In *Atta sexdens*, we found *Escovopsis* from clades I, V and also one strain (*Escovopsis* UT010) that grouped with brown-spored *Escovopsis* associated with *Apterostigma* [19], called here clade IX. Finally, the type strains of *E. microspora* and *E. moelleri*, both isolated from the same ant species *Acromyrmex subterraneus molestans* [20], grouped in clades I and V, respectively (figure 1). These results support the low host-specificity between *Escovopsis* and its leaf-cutting ant hosts, demonstrated by Taerum *et al*. [22].

The results of our phylogenetic groupings based on two nuclear markers are further supported by the presence of two morphological characters that distinguish clades I–V and VIII from clades VI, VII and IX (figure 1). Based on our samples, all *Escovopsis* strains from clades I–V and VIII have cylindrical vesicles, while all strains from clades VI and VII have globose vesicles (figure 1). All the *Escovopsis* strains from the clade mainly associated with *Apterostigma* (clade IX) analysed here also have globose vesicles. Both vesicle morphotypes were previously reported by studies that described the five known brown-spored *Escovopsis* species [15,16,20]. However, we show here that this morphological character correlates with phylogeny (i.e. we did not find strains within the same clade exhibiting different types of vesicles), suggesting that this trait is important for taxonomic studies.

Moreover, we also performed ancestral state reconstruction to test which of the two morphotypes could be considered the ancestor state using the ‘Ape’ package in R [36,37] (see Material and methods for details). Under the equal rates model, the average support for globose vesicles as the ancestral state average is 96.99% (pie-graphs in figure 1) and in none of the trees were cylindrical vesicles supported as the ancestral state. When estimated over a sample of trees from a RAxML bootstrap sample, the average support for globose vesicles as the ancestral state was 69.04%. This is lower than the support values obtained from the Bayesian sample (96.99%). One possible cause for this discrepancy is that the RAxML trees were, on average, shorter in total branch lengths than the Bayesian trees. Shorter branch lengths decrease the possibility of change along a given branch, and the trees estimated by RAxML tend to have short branch lengths near the root of the tree. These branch lengths could inflate the probability of observing cylindrical vesicles at the root.

Additional macroscopic differences (e.g. growth, time for sporulation and intensity of sporulation) were noted between strains from different clades that warrant more careful examination by taxonomists (electronic supplementary material, figure S1).

## 4. Discussion

### 4.1 *Escovopsis* occurrence in higher attine ants

Despite the efforts of attine workers to weed out pathogens from fungal gardens, alien fungi can invade attine gardens [24,40]. Some of these invaders are cosmopolitan fungi within the genera *Trichoderma*, *Fusarium* or *Syncephalastrum* that also exist outside the attine symbiosis [24]. By contrast, *Escovopsis* are fungal parasites so far only known from attine colonies and therefore are considered specialized on attine nests [14,17]. *Escovopsis* parasites have stimulated broad interest due to their potential as biological control agents of leaf-cutting ants, which are major agricultural pests throughout the tropical New World [41]. To date, the largest studies considering *Escovopsis* diversity associated with higher attine ants are those of Taerum *et al*. [22,23]. However, these authors did not deeply explore geographical areas in South America (the origin of higher attine ants) as well as the diversity of the parasite associated with non-leafcutter higher attine ants (they focused mostly on *Escovopsis* from the leafcutter ants *Acromyrmex* and *Atta*, but not from the other higher attine genera *Trachymyrmex* and *Sericomyrmex*).

Our results do not support the phylogenetic clade-to-clade correspondence between higher attine ants (i.e. between leafcutters and non-leafcutters) and their respective *Escovopsis* parasites [17,22]. *Escovopsis* associated with higher attine ants are thought to have undergone cladogenesis in parallel with the corresponding ant lineages. This was originally supported by the apparent presence of two reciprocally monophyletic *Escovopsis* clades, each associated with its own clade of higher attine ants: (i) *Escovopsis* from the leaf-cutting ants *Acromyrmex* and *Atta*, and (ii) *Escovopsis* from *Trachymyrmex*, a non-leafcutter higher attine genus [17,22]. A similar pattern of co-cladogenesis had been suggested for the mutualistic cultivar, which likewise consists of a higher attine clade, within which ‘*Attamyces*’ is cultivated by leaf-cutting ants, and the so-called ‘Trachymyces’ by *Trachymyrmex* [42]. Our phylogenetic reconstruction (figure 1) reveals exceptions to this view, as we found 12 examples of *Escovopsis* (16% of our total sampling; figure 1) infecting attine colonies other than the clade with which they were thought to be exclusively associated [17,22]. Our data suggest that, rather than tight correspondence between ant genera and particular *Escovopsis* strains, ants from any higher attine genus can share infections by closely related *Escovopsis* strains. However, it is important to mention that these patterns are not inconsistent with broad congruence between *Escovopsis* and attine ants at higher taxonomic levels [17], for example, between higher and lower attine ants other than *Apterostigma* ants (i.e. we did not find pink-, white- or yellow-spored *Escovopsis* infecting higher attine gardens; these morphotypes seem to be less derived and restricted to lower attine ants, as indicated by previous studies [17,19,21,43]).

Cultivar switching, which is known to occur among some attine species [42,44–46–51], could be one of the mechanisms for *Escovopsis* sharing between leaf-cutting and other higher attine ants. In our study, we did not sequence the cultivar of the colonies from where the *Escovopsis* strains were isolated, which would allow a test of association-specificity between the parasite and the cultivars and would permit testing of whether phylogenetic patterns of *Escovopsis* are more congruent with those of the ants or the fungal cultivars. However, comparisons with data obtained in previous studies enable us to indirectly realize that cultivar switching by ants does not seem to be the only mechanism for *Escovopsis* infection sharing. Augustin *et al*. [20] found three different *Escovopsis* species infecting the same garden within a nest of *Acromyrmex subterraneus molestans* (see the electronic supplementary material, table S2, nest no. 4 in Augustin *et al*. [20]). Like other leafcutters, this ant is supposed to cultivate a single clone of the cultivar within its colony [52]. Strains of *E. moelleri* and *E. microspora* were found in top-portions of the garden, while strains of *E. moelleri* and *E. lentecrescens* were found on the bottom of the garden. Therefore, *Escovopsis* strains infecting the same garden (i.e. a single cultivar clone) grouped in three different clades shown in figure 1 (*E. microspora*, clade I; *E. moelleri*, clade V; *E. lentecrescens*, clade VII). These clades include *Escovopsis* from leaf-cutting ants as well as *Escovopsis* thought previously to be specialized on *Trachymyrmex* ants, indicating no specificity in *Escovopsis* infections within the higher attine group (i.e. it is possible to isolate from a single ant garden *Escovopsis* strains that span a broad diversity of higher attine-associated *Escovopsis*).

One possible interpretation of the new phylogenetic pattern (figure 1) is that some *Escovopsis* clades have generalist pathogenic potential (clades I, II, V and VII), infecting different higher attine ant genera, whereas other clades (IV, VI and VIII) may be more restricted to specific ant populations or geographical regions (corresponding to endemic *Escovopsis* species). For example, although the mechanisms of *Escovopsis* transmission between ant nests is still unknown, it is possible that a generalist vector might be able to transmit *Escovopsis* from clades I, II, V and VII between nests from the majority of higher attine ants, whereas other *Escovopsis* from clades IV, VI and VIII may be transmitted by vectors more specific to particular ant clades. Other explanations for the observed association patterns include that (i) *Escovopsis* from rare clades might be more adapted to the specific ecological conditions in the regions where they are found, or (ii) some *Escovopsis* from generalist clades could have been selected to have broad host ranges (this could be driven, for example, by dispersal limitations, thus increasing the need to transition between phylogenetically distant ant or fungal lineages), while *Escovopsis* from rare clades could have evolved to overcome specific defences of specific cultivar hosts.

The presence of *Escovopsis* in the Caribbean island of Guadeloupe (strains UT001 and UT002; figure 1) is particularly interesting. *Acromyrmex octospinosus* was recently introduced to Guadeloupe and is an invasive species there [53], which makes the presence of *Escovopsis* in these colonies somewhat of a curiosity. We propose three potential hypotheses for how this relationship came to be: (i) *Escovopsis* was present in the colony that was initially introduced, suggesting that *Escovopsis* sometimes do not represent a deadly disease, because the introduced colony(ies) survived; (ii) *Escovopsis* was present on a dispersing queen that was introduced, implying that *Escovopsis* can also be vertically transmitted; or (iii) *Escovopsis* can be dispersed or vectored widely across the Neotropics to reach remote islands, indicating that *Escovopsis* is able to disperse greater distances than nest-founding queens.

Despite the observed sharing of *Escovopsis* types between leaf-cutting and non-leaf-cutting ants, the effective pathogenic potential of such strains remains unclear. Augustin *et al.* [20] isolated *E. moelleri* from many colonies of *Acromyrmex subterraneus* collected at multiple sites, which indicates this fungus is consistently associated with this ant species, and it is probably a pathogen infecting the ant’s fungal cultivars. However, in our phylogenetic analysis, *E. moelleri* grouped within a clade composed mainly of *Escovopsis* strains isolated from *Trachymyrmex* gardens (clade V, figure 1). The consistent isolations of *E. moelleri* from *A. subterraneus* colonies and its position in a *Escovopsis* clade infecting mostly *Trachymyrmex* colonies could therefore be an indication that *E. moelleri* might transit readily as a generalized symbiont between local leafcutters and non-leafcutters colonies. Nevertheless, we cannot discard the possibility of transmission between leaf-cutting and non-leaf-cutting colonies with no pathogenic action yet. Future testing on the pathogenic potential of *Escovopsis* from different clades against cultivar strains from both leaf-cutting and non-leaf-cutting ants can distinguish between these possibilities.

Gerardo *et al*. [19] were the first to document exceptions to the co-cladogenesis model when they found brown-spored *Escovopsis* (closely related to *Escovopsis* associated with higher attines) infecting *Apterostigma* ants that cultivate ‘coral fungi’ in the family Pterulaceae, which are distantly related to the higher attine cultivars. Recently, *Apterostigma* ants cultivating ‘*Attamyces*’ fungi were found in Brazil [51]. These combined findings suggest that evidence for additional switches at higher phylogenetic levels between *Escovopsis*, the attine ants and their fungal cultivars may be found in South America. Future sampling of lower attine ants, especially in Brazil, may reveal further exceptions to the co-cladogenesis model of the attine ant–cultivar–parasite symbiosis.

Emerging infectious diseases can derive from novel host–parasite interactions, for example, a host jump (i.e. parasite infection of a new host species) or spread to uninfected host populations of an existing host–pathogen association [54,55]. In some fungal–insect or fungal–plant interactions, the fungal pathogens can establish generalist infections, being able to switch between different hosts, whereas in other interactions the fungal pathogen is restricted to specific host species or populations [54,56]. Because *Escovopsis* has been found so far only associated with attine ants, it is assumed to be specialized on the ants’ cultivars [14]. The switches documented here (figure 1) are indirect indications that *Escovopsis* has the ability to spread across more phylogenetically diverse hosts than was previously known, but it is still a specific parasite linked to the fungus-growing ant system. Investigations into the life cycle of *Escovopsis* will probably reveal mechanisms underlying this attine-specificity, as well as the observed host switching across attine ant–cultivar lineages.

### 4.2 Phylogenetic and morphological diversity of higher attine *Escovopsis*

*Escovopsis* diversity could be affected by climate and other environmental conditions, and may therefore follow a latitudinal diversity gradient. Four colonies of *Atta cephalotes* from Camacan, Bahia, Brazil (figure 2; electronic supplementary material, table S1) contained *Escovopsis* from three different clades (figure 1), including clade IV, currently known only from that site. Augustin *et al*. [20] also found *Escovopsis* from three different clades (I, V and VII) associated with a single colony of *Acromyrmex* in Viçosa (site no. 23 in figure 2), Brazil. By contrast, our sampling of *Acromyrmex* species from southern Brazil only revealed *Escovopsis* strains from clade I. This preliminary analysis therefore suggests a somewhat reduced genetic diversity at higher latitudes, but more comprehensive collections across latitudinal gradients will be needed to firmly establish this latitudinal pattern.

Until recently, *Escovopsis* was characterized into two types, ‘weberi morphology’ and ‘aspergilloides morphology’, corresponding to cylindrical and globose vesicles, respectively [15,16]. However, Augustin *et al*. [20] were the first to recognize new species within each of these two morpho-groups when describing *E. microspora*, *E. moelleri* and *E. lentecrescens*. Our analyses (figure 1) expand on Augustin *et al*.’s [20] work by identifying additional *Escovopsis* clades, as well as several new strains or species within the two previously known morpho-groups. To avoid taxonomic confusion, future studies should therefore consider using either ‘globose’ or ‘cylindrical’ vesicles for a morphological classification for this group (instead of using ‘weberi morphology’ and ‘aspergilloides morphology’).

Ancestral character-state reconstruction indicated that globose vesicle appears to be the ancestral state relative to the phylogenetically derived cylindrical vesicles, being present in *Escovopsis* that commonly infect *Apterostigma* gardens and in *Escovopsis* UT010 (clade IX; figure 1 and electronic supplementary material figure S2, prob = 0.97). Therefore, two main scenarios can explain these observed phylogenetic patterns (figure 1): (i) cylindrical vesicles arose in the ancestor of clade VIII and clades I–VII and after that, independent reversions to globose vesicles occurred for clades VI and VII, while all other clades maintained the derived character; (ii) cylindrical vesicles arose independently twice, once in clade VIII and once in the ancestor of all derived clades I–V. Our data support the second scenario more strongly (figure 1; electronic supplementary material, figure S2). However, future sampling of *Apterostigma* ants from Brazil (especially searching for brown-spored *Escovopsis*) will be necessary to better understand the morphological evolution and evolutionary transitions of associations with globose versus cylindrically vesicled *Escovopsis*. Such studies can then also address the predicted additional switches between *Escovopsis* infecting higher attine ants and the lower attine *Apterostigma*, as discussed above.

In addition to the defining morphological differentiation between globose versus cylindrical vesicles, we found that strains from different *Escovopsis* clades can exhibit very distinct growth, time of sporulation and sporulation intensity when cultivated under the same conditions (electronic supplementary material, figure S1). Such differences, as well as more detailed microscopic analyses (e.g. of conidia structure and size or conidiophore morphology) will be necessary to inform future taxonomy. The phylogenetic patterns documented here (figure 1) hint at an underappreciated diversity of *Escovopsis* and the complexity of its evolutionary history; and will provide a firm basis for future taxonomic and ecological studies of *Escovopsis* fungi associated with higher attine ants.

## Supplementary Material

supp_mat_RSOS_reviewed_vfinal.docx This file contains Figures S1 and S2, in addition to Table S1.

## Data accessibility

DNA sequences: GenBank accessions KM817043–KM817173. For details, see the electronic supplementary material, table S1. Phylogenetic data, including alignments: TreeBASE accession number S16556 (http://purl.org/phylo/treebase/phylows/study/TB2:S16556).
