# Supplementary material for: Shared Escovopsis parasites between leaf-cutting and non-leaf-cutting ants in the higher attine fungus-growing ant symbiosis
Source: R Soc Open Sci. 2015 Sep 30;2(9):150257. doi: 10.1098/rsos.150257 (PMC4593684; doi:10.1098/rsos.150257)
Supplement: supp_mat_RSOS_reviewed_vfinal.docx This file contains Figures S1 and S2, in addition to Table S1. [file rsos150257supp1.docx]

**Supplementary material**

Shared *Escovopsis* infections between leaf-cutting and non-leaf-cutting ants in the higher-attine fungus-growing ant symbiosis

Lucas A. Meirelles, Scott E. Solomon, Mauricio Bacci Jr., April M. Wright, Ulrich G. Mueller, Andre Rodrigues

**Fig. S1.** Morphological aspects of *Escovopsis*. Conditions were 7 seven days of growth on PDA medium at 25 ºC. I-IX each represent growth of one strain of each clade displayed in Fig. 1, indicated by the colors used on the edge of each photo. Growth, sporulation time and conidia maturation (i.e. sporulation for brown-spored *Escovopsis* is characterized by the white young conidia, becoming brown when mature) differ between strains.


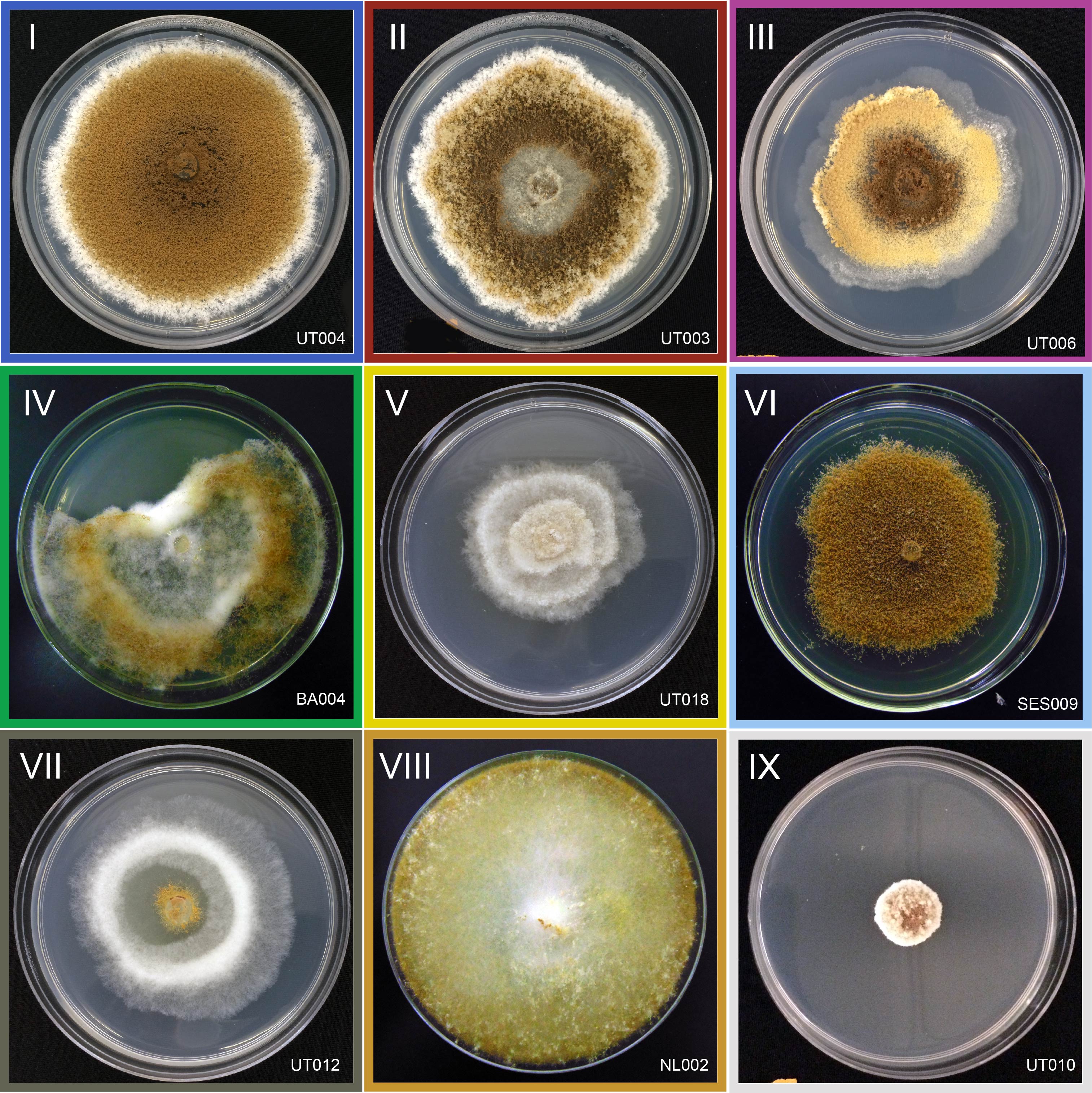


**Fig. S2.** Ancestral state reconstruction for vesicle type of *Escovopsis* displayed on the Bayesian consensus tree. Probabilities are shown in pie-graph format where red indicates globose vesicles and blue indicates cylindrical vesicles. The tree is the same displayed in Fig. 1.


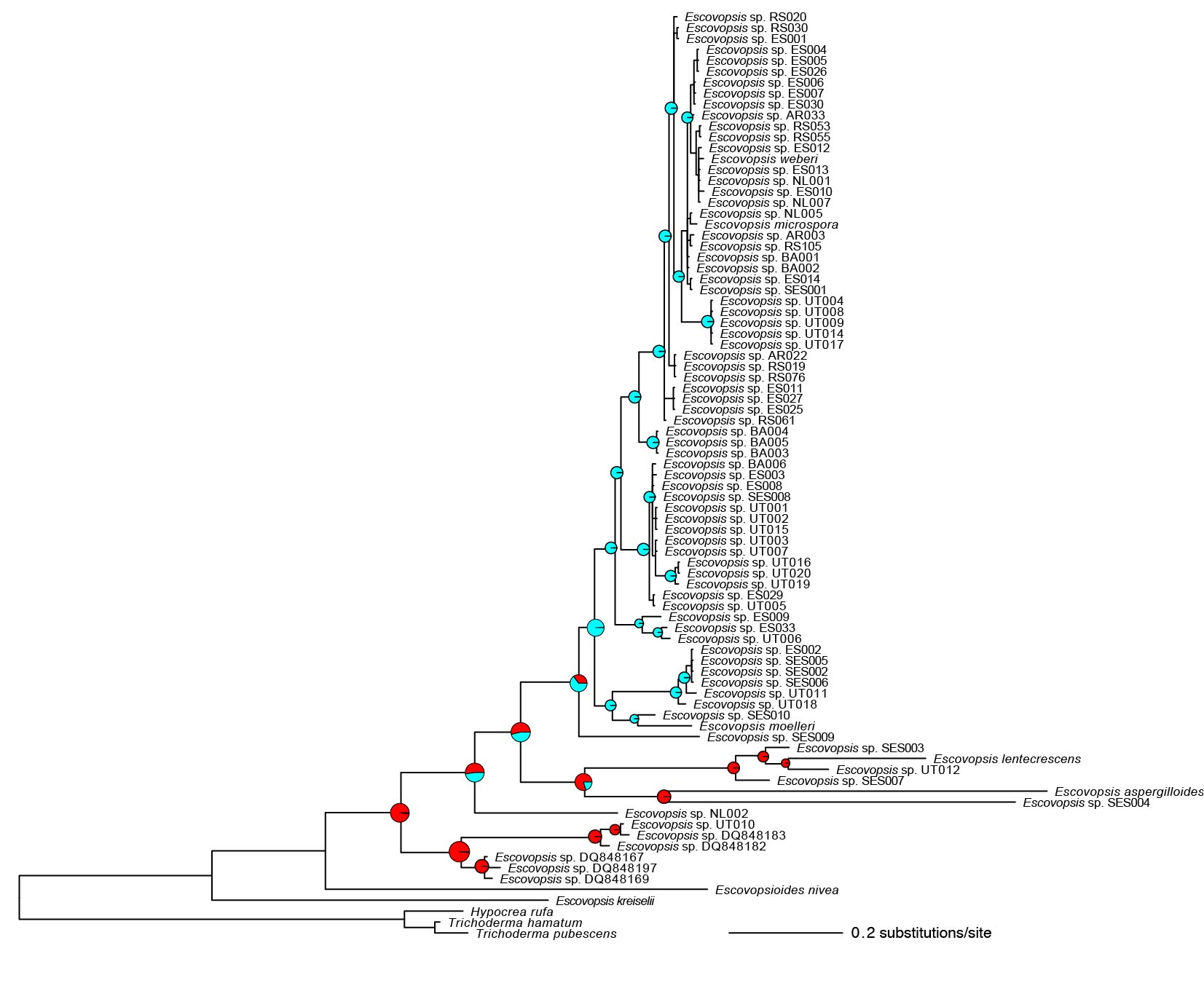


Table S1. *Escovopsis* strains analyzed in this study. Data from described species of brown-spored *Escovopsis* were derived from previous studies [16, 20]. It is also informed molecular data for *Escovopsioides nivea* and *Escovopsis kreiselii* [20, 21] as well as other brown-spored *Escovopsis* strains derived from Gerardo et al. [19]. Data from Hypocreaceae species used as outgroup are also shown.

| STRAIN ID | ANT | NEST ID | SOURCE | LOCAL | LOCAL ID | *tef1* | ITS |
| --- | --- | --- | --- | --- | --- | --- | --- |
| NL001^1^ | *Atta capiguara* | N66 | Midden | Botucatu - SP, Brazil | 1 | KM817142 | KM817072 |
| NL002^1^ | *Atta capiguara* | N66 | Fungus garden | Botucatu - SP, Brazil | 1 | KM817143 | KM817073 |
| NL005^2^ | *Atta sexdens* | N68 | Fungus garden | Botucatu - SP, Brazil | 1 | KM817144 | KM817074 |
| NL007^2^ | *Atta sexdens* | N68 | Fungus garden | Botucatu - SP, Brazil | 1 | KF240730 | KM817075 |
| ES002 | *Atta sexdens* | N-Ale | Fungus garden | Rio Claro - SP, Brazil | 2 | KM817123 | KM817053 |
| ES011 | *Atta sexdens* | 6B | Fungus garden | Corumbataí - SP, Brazil | 3 | KM817132 | KM817062 |
| ES012 | *Atta sexdens* | 13B | Fungus garden | Corumbataí - SP, Brazil | 3 | KM817133 | KM817063 |
| ES013 | *Atta sexdens* | 1 | Fungus garden | Corumbataí - SP, Brazil | 3 | KM817134 | KM817064 |
| ES014 | *Atta sexdens* | 4 | Fungus garden | Corumbataí - SP, Brazil | 3 | KM817135 | KM817065 |
| ES003 | *Atta cephalotes* | SES040121-01 | Fungus garden | Frei Caneca - PE, Brazil | 4 | KM817124 | KM817054 |
| ES005 | *Atta cephalotes* | SES040129-06 | Fungus garden | Alta Floresta - MT, Brazil | 5 | KM817126 | KM817056 |
| ES009 | *Atta cephalotes* | SES040220-01 | Fungus garden | Carreiro da Varzea - AM, Brazil | 6 | KM817130 | KM817060 |
| ES033 | *Atta cephalotes* | CTL110912-05 | Fungus garden | Parauapebas - PA, Brazil | 7 | KM817141 | KM817071 |
| RS105 | *Atta laevigata* | AOMB170904-02 | Fungus garden | T. de Santa Bárbara - SP, Brazil | 8 | KM817145 | KM817083 |
| BA001 | *Atta cephalotes* | BMSR120702-01(FL1) | Fungus garden | Camacan - BA, Brazil | 9 | KM817116 | KM817046 |
| BA002 | *Atta cephalotes* | BMSR120803-02(CA7) | Fungus garden | Camacan - BA, Brazil | 9 | KM817117 | KM817047 |
| BA003^3^ | *Atta cephalotes* | BMSR120803-01(CA10) | Fungus garden | Camacan - BA, Brazil | 9 | KM817118 | KM817048 |
| BA004^4^ | *Atta cephalotes* | BMSR120703-01(FL5) | Fungus garden | Camacan - BA, Brazil | 9 | KM817119 | KM817049 |
| BA005^4^ | *Atta cephalotes* | BMSR120703-01(FL5) | Fungus garden | Camacan - BA, Brazil | 9 | KM817120 | KM817050 |
| BA006^3^ | *Atta cephalotes* | BMSR120803-01(CA10) | Fungus garden | Camacan - BA, Brazil | 9 | KM817121 | KM817051 |
| ES004 | *Acromyrmex* sp. | SES040125-08 | Fungus garden | Camacan - BA, Brazil | 9 | KM817125 | KM817055 |
| ES006 | *Ac. coronatus* | SES040130-11 | Fungus garden | Alta Floresta - MT, Brazil | 6 | KM817127 | KM817057 |
| ES007 | *Ac. coronatus* | SES040131-07 | Fungus garden | Alta Floresta - MT, Brazil | 6 | KM817128 | KM817058 |
| ES008 | *Acromyrmex* sp. | SES040216-02 | Fungus garden | Santarem - PA, Brazil | 10 | KM817129 | KM817059 |
| ES010 | *Ac. landolti* | - | Fungus garden | Rio Claro - SP, Brazil | 2 | KM817131 | KM817061 |
| ES025 | *Ac. balzanii* | AR05 | Fungus garden | Botucatu - SP, Brazil | 1 | KM817136 | KM817066 |
| ES027 | *Ac. rug. rugosus* | - | Midden | Rio Claro - SP, Brazil | 2 | KM817138 | KM817068 |
| RS019 | *Ac. ambiguus* | AOMB060904-04 | Fungus garden | Nova Petropolis - RS, Brazil | 11 | EU082802 | KM817076 |
| RS020 | *Ac. laticeps* | AOMB060904-05 | Fungus garden | Nova Petropolis -RS, Brazil | 11 | EU082803 | KM817077 |
| RS030 | *Ac. lundi* | AOMB080904-02 | Fungus garden | São Marcos - RS, Brazil | 12 | EU082795 | KM817078 |
| RS053 | *Ac. lundi* | AOMB110904-02 | Fungus garden | Chuvisca - RS, Brazil | 13 | EU082797 | KM817079 |
| RS055 | *Ac. heyeri* | AOMB110904-07 | Fungus garden | Chuvisca - RS, Brazil | 13 | EU082796 | KM817080 |
| RS061 | *Ac. heyeri* | AOMB110904-15 | Fungus garden | Pelotas - RS, Brazil | 14 | EU082799 | KM817081 |
| RS076 | *Ac. coronatus* | AOMB130904-04 | Fungus garden | Vacaria - RS, Brazil | 15 | EU082801 | KM817082 |
| SES008 | *Acromyrmex* sp. | SES081007-01 | Fungus garden | Faz. São Sebastião - RO, Brazil | 16 | KM817152 | KM817091 |
| AR003 | *Ac. balzanii* | AR110511-01 | Fungus garden | Ilhéus - BA, Brazil | 17 | KM817113 | KM817043 |
| AR022 | *Acromyrmex* sp. | AR110515-01 | Fungus garden | Camacan - BA, Brazil | 9 | KM817114 | KM817044 |
| AR033 | *Acromyrmex* sp. | ARFV6110517-01 | Fungus garden | Camacan - BA, Brazil | 9 | KM817115 | KM817045 |
| ES001 | *Trachymyrmex* sp. | TR-117 | Fungus garden | Rio Claro - SP, Brazil | 2 | KM817122 | KM817052 |
| ES026 | *Trachymyrmex* sp. | ARTD030908-02 | Fungus garden | Rio Claro - SP, Brazil | 2 | KM817137 | KM817067 |
| ES029 | *Trachymyrmex* sp. | WGPM091021-01 | Fungus garden | Palmas - TO, Brazil | 18 | KM817139 | KM817069 |
| ES030 | *Trachymyrmex* sp. | AR091020-01 | Fungus garden | Palmas - TO, Brazil | 18 | KM817140 | KM817070 |
| SES001 | *Trachymyrmex* sp. | SES080402-03 | Fungus garden | Rio Claro - SP, Brazil | 2 | KM817146 | KM817084 |
| SES002 | *Trachymyrmex* sp. | SES080408-02 | Fungus garden | Fazenda Pau Brasil - GO, Brazil | 19 | KM817147 | KM817085 |
| SES003 | *Trachymyrmex* sp. | CTL080820-02 | Fungus garden | E.E.Panga, Uberlândia - MG, Brazil | 20 | KM817148 | KM817086 |
| SES005 | *Trachymyrmex* sp. | SES080922-03 | Fungus garden | E.E.Panga, Uberlândia - MG, Brazil | 20 | KF240731 | KM817088 |
| SES006 | *T. dichrous* | SES080922-02 | Fungus garden | E.E.Panga, Uberlândia - MG, Brazil | 20 | KM817150 | KM817089 |
| SES007 | *Trachymyrmex* sp. | SES080921-03 | Fungus garden | E.E.Panga, Uberlândia - MG, Brazil | 20 | KM817151 | KM817090 |
| SES009 | *Trachymyrmex* sp. | SES081108-04 | Fungus garden | Palmeiras - BA, Brazil | 21 | KM817153 | KM817092 |
| SES010 | *T. diversus* | SES090109-04 | Fungus garden | Camp 41, Manaus - AM, Brazil | 22 | KM817154 | KM817093 |
| SES004 | *S. luederwaldti* | CTL080825-02 | Fungus garden | E.E.Panga, Uberlândia - MG, Brazil | 20 | KM817149 | KM817087 |
| *Escovopsis weberi* | Ant nest | - | - | Brazil | - | AY172623 | KF293286 |
| *Escovopsis microspora* | *Ac. sub. molestans.* | **-** | Fungus garden | Viçosa - MG, Brazil | 23 | KJ935030 | JQ815076 |
| *Escovopsis moelleri* | *Ac. sub. molestans.* | **-** | Fungus garden | Viçosa - MG, Brazil | 23 | JQ855712 | JQ815077 |
| *Escovopsis lentecrescens* | *Ac. sub. subterraneus* | **-** | Fungus garden | Viçosa - MG, Brazil | 23 | JQ855714 | JQ815079 |
| UT001 | *Ac. octospinosus* | ASM031224-02 | Fungus garden | Caribbean island of Guadeloupe | 24 | KM817155 | KM817094 |
| UT002 | *Acromyrmex* sp. | ASM021224-07 | Fungus garden | Caribbean island of Guadeloupe | 24 | KM817156 | KM817095 |
| UT003 | *Acromyrmex* sp. | CC020602-06 | Fungus garden | Gamboa, Panama | 25 | KM817157 | KM817096 |
| UT004^5^ | *Atta colombica* | NMG010319-24 | External dump | Gamboa, Panama | 25 | KM817158 | KM817097 |
| UT005 | *Acromyrmex* sp. | SP030327-01 | Fungus garden | Misiones, Argentina | 26 | KM817159 | KM817098 |
| UT006 | *Atta cephalotes* | CC010324-14 | Fungus garden | Gamboa, Panama | 25 | KM817160 | KM817099 |
| UT007 | *Atta colombica* | NMG010319-25 | External dump | Gamboa, Panama | 25 | KM817161 | KM817100 |
| UT008 | *Atta colombica* | NMG010319-22 | External dump | Gamboa, Panama | 25 | KM817162 | KM817101 |
| UT009^5^ | *Atta colombica* | NMG010319-24 | External dump | Gamboa, Panama | 25 | KM817163 | KM817102 |
| UT010 | *Atta sexdens* | CC020529-01 | Fungus garden | Coclecito, Panama | 27 | KM817164 | KM817103 |
| UT011 | *Trachymyrmex* sp. | SES020522-02 | Fungus garden | Gamboa, Panama | 25 | KM817165 | KM817104 |
| UT012 | *Trachymyrmex* sp. | AL030106-09 | Fungus garden | "Canal Zone", Panama | 28 | KM817166 | KM817105 |
| UT014 | *Atta colombica* | UGM030106-16 | Fungus garden | Darien, Panama | 29 | KM817167 | KM817106 |
| UT015 | *Atta colombica* | UGM030106-17 | Fungus garden | Darien, Panama | 29 | KM817168 | KM817107 |
| UT016 | *Trachymyrmex* sp. | JS030114-01 | Fungus garden | Palenque, Mexico | 30 | KM817169 | KM817108 |
| UT017 | *Atta colombica* | NMG010319-22 | External dump | Gamboa, Panama | 25 | KM817170 | KM817109 |
| UT018 | *Trachymyrmex* sp. | SP011102-01 | Fungus garden | Gamboa, Panama | 25 | KM817171 | KM817110 |
| UT019 | *Atta* *cephalotes* | SES030113-01 | Fungus garden | Palenque, Mexico | 30 | KM817172 | KM817111 |
| UT020 | *Trachymyrmex* sp. | JS030113-01 | Fungus garden | Palenque, Mexico | 30 | KM817173 | KM817112 |
| *Escovopsis aspergilloides* | *Trachymyrmex ruthae* | - | Fungus garden | Trinidad and Tobago | 31 | AY172632 | KF293287 |
| *Escovopsioides nivea* | *Ac. sub. subterraneus* | - | Fungus garden | Viçosa - MG, Brasil | 23 | JQ855713 | JQ815078 |
| *Escovopsis kreiselii* | *Mycetophylax morschi* | AR090306-01 | Fungus garden | Florianópolis, SC, Brasil | - | KJ808766 | KJ808767 |
| Brown-spored *Escovopsis* derived from Gerardo et al. [19] | | | | | | | |
| *Escovopsis* sp. | *Apterostigma dentigerum* | AGH020629-02 | Fungus garden | Costa Rica | - | DQ848167 | - |
| *Escovopsis* sp. | *Apterostigma* sp. | AGH030627-03 | Fungus garden | Ecuador | - | DQ848197 | - |
| *Escovopsis* sp. | *Apterostigma dentigerum* | AGH020709-10 | Fungus garden | Costa Rica | - | DQ848169 | - |
| *Escovopsis* sp. | *Apterostigma dentigerum* | UGM020531-01 | Fungus garden | Panama | - | DQ848183 | - |
| *Escovopsis* sp. | *Apterostigma* sp. | AGH030627-08 | Fungus garden | Ecuador | - | DQ848182 | - |
| Other species of Hypocreaceae used as outgroup | | | | | | | |
| *Hypocrea rufa* | - | - |  | - | - | AF534585 | X93980 |
| *Trichoderma hamatum* | - | - |  | - | - | AF534620 | EU280124 |
| *Trichoderma pubescens* | - | - |  | - | - | AF534624 | NR_077179 |

Abbreviations: *Ac.* = *Acromyrmex*; *rug.* = *rugosus*; *sub.* = *subterraneus*; *S.* = *Sericomyrmex*; *T.* = *Trachymyrmex*.

^1,2,3,4,5^ Numbers after the strain ID indicate *Escovopsis* isolated from the same nest.
